# Pilot Study of Intelligent Office Blood Pressure Measurement Model in Shanghai, China, 2022

**DOI:** 10.5334/gh.1344

**Published:** 2024-08-21

**Authors:** Guoli Wu, Qinghua Yan, Fernando Martínez-García, Dinesh Neupane, Yuheng Wang, Fei Wu, Cui Wu, Barbara Lee Smith, Yan Shi, Minna Cheng

**Affiliations:** 1 Global Health, Project HOPE, Shanghai, China; 2 Division of Non-communicable Diseases and Injury Control and Prevention, Shanghai Municipal Center for Disease Control and Prevention, Shanghai, China; 3 Internal Medicine Department, Clinical Hospital of Valencia, Valencia, Spain; 4 Cardio-metabolic and Renal Risk Research Group, Research Institute of the Clinical Hospital of Valencia (INCLIVA), Valencia, Spain; 5 Medicine Department, University of Valencia, Valencia, Spain; 6 Department of International Health, Bloomberg School of Public Health, Johns Hopkins University, Baltimore, Maryland, USA; 7 Department of Non-communicable Diseases Control and Prevention, Baoshan District Center for Disease Control and Prevention, Shanghai, China; 8 Global Health, Project HOPE, Washington, DC, USA

**Keywords:** Blood Pressure, Hypertension, Office Blood Pressure Measurement, Community Health Centers, End-digit, Management Information Systems

## Abstract

**Introduction:**

An intelligent office blood pressure measurement (IOBPM) model for community-based hypertension management was piloted in Shanghai, China, to overcome the conventional blood pressure management (CBPM) model’s deficiencies.

**Methods:**

We selected adults aged 35–89 years who were being treated and managed for hypertension in two community health centers for the IOBPM and CBPM models. The IOBPM model consisted of two or three consecutive blood pressure (BP) measurements using a pre-programmed and validated automatic device. The BP data for the CBPM model were obtained from the routine follow-up records of hypertensive patients and derived from the Shanghai Non-communicable Diseases Management Information System. Subjects in the IOBPM model were selected by a simple random sampling method, and propensity score matching was used to select a comparable control population from the CBPM model based on important covariables. The BP levels, end-digit preferences, frequency distribution, and BP control were compared between the two models.

**Results:**

We selected 2,909 patients for the IOBPM model and 5,744 for the CBPM model. The systolic BP in the CBPM model was 12.3 mmHg lower than in the IOBPM model. In the CBPM model, there were statistically significant end-digit preferences (*P* < 0.001), with zero being the most reported end-digit (23.3% for systolic BP and 27.7% for diastolic BP). There was no significant end-digit preference in the IOBPM model. Certain BP values below 140/90 mmHg in the CBPM model were more frequent, while the IOBPM model showed a normal distribution. The BP control in the CBPM model was significantly higher than the IOBPM model (*P* < 0.001).

**Conclusion:**

The IOBPM model appears to overcome the deficiencies of the CBPM model, leading to more accurate and reliable BP measurements.

## Introduction

Shanghai has the most elderly population of aged over 60 individuals in China (23.4%), according to the data of the seventh National Population Census in 2020 (1). With an aging population, hypertension management among older adults is increasingly in demand (2). The hypertension prevalence in Shanghai (29.1%) among those aged over 18 was higher than the average in China (23.2%) in 2015 (3). More than 200 community health centers (CHCs) in Shanghai provide hypertension management services to over two million people through the chronic non-communicable diseases (NCDs) management information system of the Shanghai Municipal Center for Disease Control and Prevention (SCDC).

The current conventional blood pressure management (CBPM) model faces several challenges: lack of a standardized method of blood pressure (BP) measurement; manual entry of BP readings; use of patient self-reporting home BP measurements (HBPM); frequent use of end-digit preference when using mercury sphygmomanometers; lack of adherence to the national hypertension guidelines; and lack of variation of BP measurements between three-month follow-up visits.

Unreliable BP measurements place patients at risk of cardiovascular events or secondary side effects (4,5). To address this issue, an innovative methodology for BP measurements was developed, the intelligent office blood pressure measurement (IOBPM) model, based on a previously published study in Shanghai’s Minhang district (6). The IOBPM model includes the hardware (automated electronic sphygmomanometer validated to international standards, automated equipment for data acquisition and display), software (information system for patient recognition, automatic data capture and transmission) and quality control system (7,8).

Identifying and promoting standardized BP measurement procedures using automated equipment to capture the data could provide quality data for hypertension management, which could be shared confidently with policymakers, implementors, and patients. We aimed to pilot the IOBPM model and compare its results with the currently in-use CBPM model to evaluate whether BP data quality can be improved.

## Methods

### Study design

This pilot study, to compare BP levels, the end-digit preferences, frequency distribution, and BP control between the two BP measurement models, was conducted from November 2021 to January 2022 in Shanghai, China. Briefly, the IOBPM model automatically performs two or three consecutive BP measurements with a pre-set interval of one minute using the electronic sphygmomanometers Omron HBP-1100U (9) and automatically calculates the average, considered to be the patient’s true BP value. The data acquisition equipment is programmed to guide the patients to take their own BP measurements in a specific area/room in the CHCs.

The CBPM model, in use before and during the pilot test, had no automatic data transmission system, no dedicated area for BP measurement at the CHCs, and no mandatory office BP measurements, but allowed for either an office visit for BP measurements or telephone follow-up to collect self-reported BP by patients who used the HBPM.

### Study population and site

We selected two townships, Luodian and Luojing, of Baoshan district in Shanghai based on location, population size, economic level, and willingness to cooperate, that were concurrently operating both the IOBPM and CBPM models. We selected adult subjects aged 35–89 years who were being treated and managed for hypertension in these two CHCs.

### Sampling methods and sample size

Data for the IOBPM model came from the baseline data of a randomized community intervention trial aimed to compare the hypertension control between the IOBPM and CBPM models at the two selected CHCs. The sample size for the trial was calculated based on the assumption of a 20% increase in BP control from 36.56%, which was from Shanghai NCDs epidemiological survey in 2018 (10), to 43.87% with an estimated non-response of 20% at the baseline and a lost-to-follow-up of 20%. The final sample size was 3,600 patients for the two CHCs. Using simple random sampling, 2,991 patients (83% response) were selected for inclusion in the IOBPM model.

For the CBPM model, the BP data we used for the comparison was extracted from the Shanghai NCDs management information system. All the hypertensive patients aged 35–89 years who had one BP follow-up recording in the system within three months before the pilot were selected. We excluded any patient who had BP measurements recorded using the IOBPM model. A total of 14,726 patients with hypertension were selected in the CBPM model.

### Variables and measurements

The variables of interest were BP levels, end-digit preferences, frequency distribution, and BP control. We described BP levels using means (with standard deviations); end-digit preferences; and BP control were described as proportions. For frequency distribution, we presented the systolic BP (SBP) and diastolic BP (DBP) percentage values. The end-digit values (zero to nine) were extracted from the SBP and DBP values, and we defined the end-digit preferences as the end-digits significantly higher than other end-digits. The BP control was defined as SBP <140 mmHg and DBP <90 mmHg. Smoking status was defined as daily tobacco smoker, occasional smoker, or non-smoker. Current Smokers included daily tobacco smokers and occasional smokers. Current alcohol consumption was defined as drinking at least once in the past year.

### Data collection

In the IOBPM model, quality control involved training all CHC health care professionals (HCPs) for IOBPM protocols and procedures. This training emphasized correct participant positioning, appropriate cuff sizing, and the importance of a 5–10 minute rest period before taking two or three consecutive seated BP measurements. The quality control team oversaw both the training and its implementation.

All patients in the IOBPM model participated in a face-to-face close-ended survey by trained HCPs at the two CHCs. Anthropometric measurements (weight and height) and BP data were obtained following the protocol of the IOBPM model described previously. The SCDC designed the questionnaire, focused on social demographic factors (age, sex, education level, and lifestyle habits). For the CBPM model, all variables, including demographics (age, sex, education level, and lifestyle habits) were extracted from the NCDs management information system.

Before the BP measurement, patients under the IOBPM model were instructed to remain comfortable and quietly seated for at least five minutes. The IOBPM had three connected components: an identity recognition device, a validated automated electronic sphygmomanometer, and software for pre-programming the IOBPM procedures. The BP monitor and the identity recognition device were connected with a computer that had the software installed. The computer software controls the entire measurement process, including identity verification, measurement prompts, intervals between measurements, completion prompts, display of BP readings, and automatic data storage and transmission. At the beginning of the measurement, the identity recognition device automatically identified patients when they swiped their ID or medical insurance card. The computer programme then guided them in taking their BP measurements. The first measurements were taken on both arms; the average of the two readings was automatically used as the patient’s true BP value. If the difference between the two SBP or DBP measurements exceeded five mmHg, or if the patient had an irregular pulse, a third measurement was taken, and the average of the three readings was calculated. Blood pressure values were automatically transmitted to the health information platform, which connected the electronic health records (EHR) of CHCs, the NCDs management information system, and other clinical practice and BP management platforms. During the measurement, one or two HCPs were present to ensure the correct cuff selection and implementation of the protocol.

In the CBPM model during routine office visits, primary care physicians (PCPs) conducted one-time BP measurements. If the PCPs were not available, or if the patients were unable to attend in person, telephone follow-ups were made to collect self-reported BP data from those using HBPM. HCPs manually entered these data into the NCDs management information system, including both routine office BP measurements and self-reported HBPM data without differentiation.

### Data analysis

Data from the IOBPM model questionnaires were double entered in Epi-data software. All databases were connected and merged using a unique ID. A standardized data cleaning process included eliminating duplicate data, judging missing values, and logical errors. We checked the missing values and logical errors with the selected CHCs and updated the database. All data required for the CBPM model was obtained from the NCDs information system.

All continuous variables were checked for normality. Data were expressed as mean ± SD/median interquartile range (IQR) or as proportions for continuous and categorical variables, respectively. Comparisons between the two models were examined using the Wilcoxon rank sum test (BP level) and the Chi-square (end-digit preferences and BP control). We applied the IQR method (k = 1.5) to exclude the outliers in both models. To address the imbalanced distribution of sex, age, education level, body mass index (BMI), smoking and drinking between the two models, we used propensity score matching (PSM) with a calliper value of 0.02. This matched the databases at a 1:2 ratio, based on variables showing highly significant differences between the two models (*P* < 0.001).

The BP control across age groups was analysed for each model using the Cochran-Armitage test for trends. We assumed that the frequency of the end-digit values of BP measurements was 10% for digits 0 through 9. The end-digit preferences in SBP and DBP within both models were examined using the Chi-square test. We used a box plot and histogram to show the distribution of SBP readings in the two models. Statistical analyses were performed using SAS (version 9.4; SAS Institute Inc, Cary, NC). Figures were created using R software (4.2.2). A two-sided *P* < 0.05 was considered statistically significant.

### Ethical approval

The Institutional Review Board (IRB) of SCDC approved the study (2021–109). All participants provided written informed consent via an active permission protocol based on the SCDC requirements. For the CBPM model, as data were obtained retrospectively from the NCD management database, the SCDC IRB approved an exemption for informed consent (2023–40).

## Results

### Study population

After the exclusion of the outliers and the PSM, the IOBPM model included 2,909 patients; 54.5% (1,585) were female. The mean age was 69.6 ± 7.8 years. The CBPM model group included 5,744 patients; 54.7% (3,140) were female. The mean age was 67.9 ± 8.4 years. As expected, because of the PSM, all the characteristics were similar between the two models (Table 1).

**Table 1:** Characteristics of hypertensive patients.


CHARACTERISTIC	IOBPM		CBPM		*P^a^*

	n	%	n	%	

Sex					0.87

Male	1324	45.5	2604	45.3	

Female	1585	54.5	3140	54.7	

Age group (years)					0.72

35–59	324	11.2	675	11.7	

60–69	1141	39.2	2233	38.9	

70–79	1196	41.1	2321	40.4	

80–89	248	8.5	515	9.0	

Education					0.62

Primary and below	1189	40.9	2396	41.7	

Junior and High school	1649	56.7	3197	55.7	

College and above	71	2.4	151	2.6	

BMI^b^					0.62

<23.9	767	26.4	1535	26.7	

24–27.9	1412	48.5	2823	49.2	

≥28	730	25.1	1386	24.1	

Current tobacco smoker	584	20.1	1144	19.9	0.86

Current alcohol consumption	580	20.0	1130	19.7	0.77


Abbreviations: IOBPM, intelligent office blood pressure measurement; CBPM, conventional blood pressure management; BMI, body-mass index. ^a^ Differences between IOBPM and CBPM models were determined by χ^2^ tests. ^b^ BMI is the weight in kilograms divided by the square of the height in meters.

### BP levels

**Figure 1:**
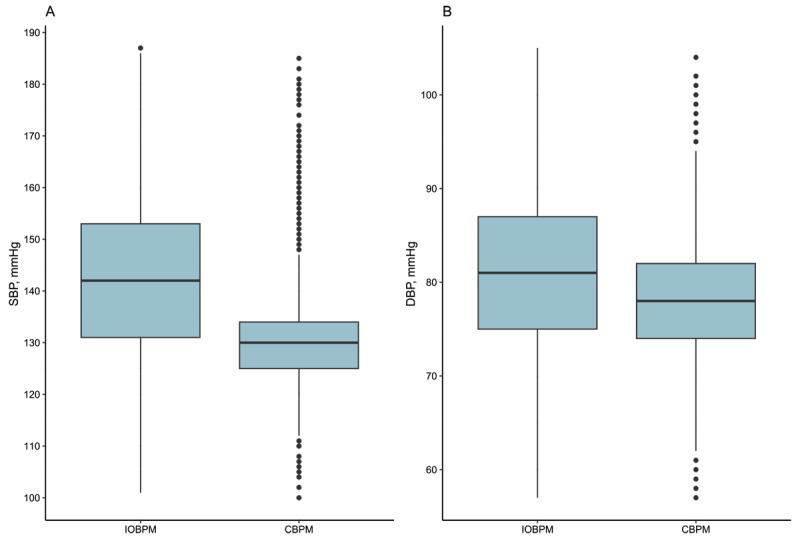
Box-plot for the comparison of systolic blood pressure and diastolic blood pressure. Abbreviations: IOBPM, intelligent office blood pressure measurement; CBPM, conventional blood pressure management; SBP, systolic blood pressure; DBP, diastolic blood pressure.

The IOBPM model had a mean SBP and DBP of 142.5 ± 15.7 mmHg and 81.0 ± 8.8 mmHg, respectively, whereas the CBPM model had a mean Systolic BP and DBP of 130.2 ± 8.7 mmHg and 77.6 ± 6.3 mmHg, respectively. Systolic BP and DBP in the CBPM model were significantly lower than in the IOBPM model (mean differences for SBP of –12.3 mmHg and DBP of –3.4 mmHg, *P* < 0.001) (Figure 1).

### End-digit preferences

**Figure 2:**
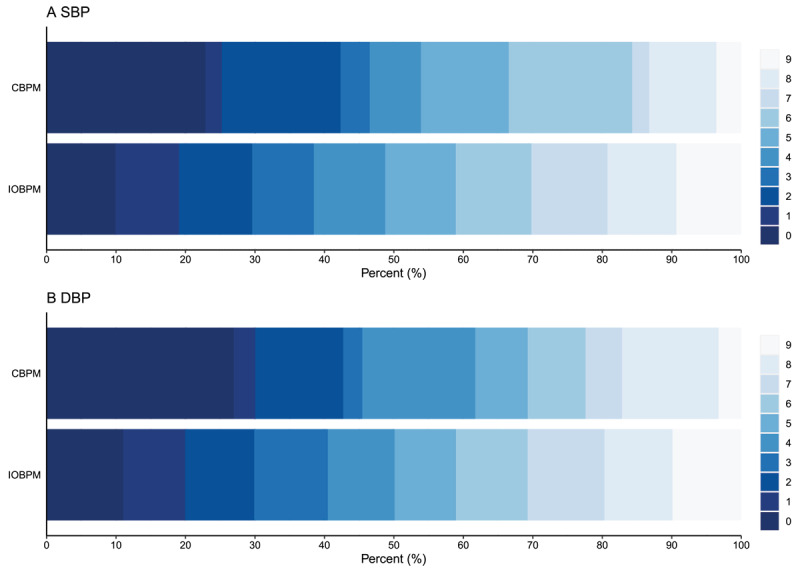
The end-digit distribution of systolic blood pressure and diastolic blood pressure. Abbreviations: IOBPM, intelligent office blood pressure measurement; CBPM, conventional blood pressure management; SBP, systolic blood pressure; DBP, diastolic blood pressure.

The frequency range of end-digit values in both SBP and DBP between the IOBPM and CBPM models was statistically different (*P* < 0.001). Within the CBPM model, the frequency range of end-digit values was significantly different, with a preference for ‘zero, two, four, six, eight’ (*P* < 0.001). The most frequent end-digit was ‘zero’ (23.3% in SBP and 27.7% in DBP readings), followed by ‘six’ (17.7%) and ‘two’ (17.3%) for SBP readings, and ‘four’ (16.9%) and ‘two’ (13.3%) for DBP readings. In contrast, in the IOBPM model, there were no significant differences regarding the end-digits of either the SBP or DBP (*P* = 0.64 and *P* = 0.67, respectively) (Figure 2).

### Frequency distribution

**Figure 3:**
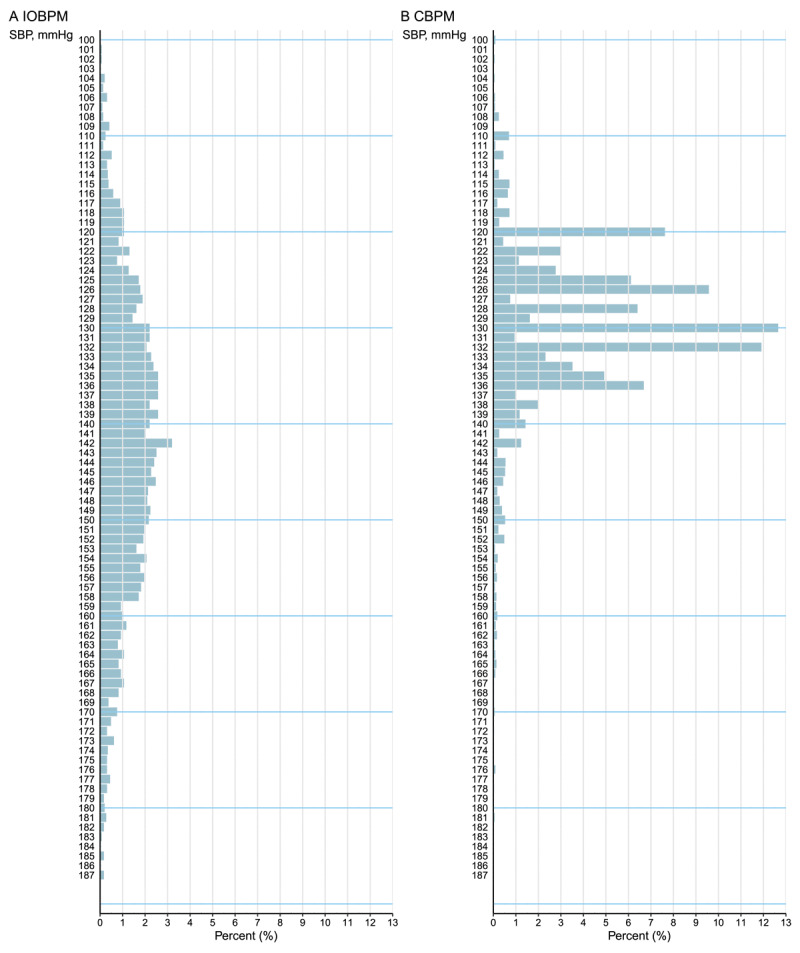
The comparison of systolic blood pressure measurements. Abbreviations: IOBPM, intelligent office blood pressure measurement; CBPM, conventional blood pressure management; SBP, systolic blood pressure.

**Figure 4:**
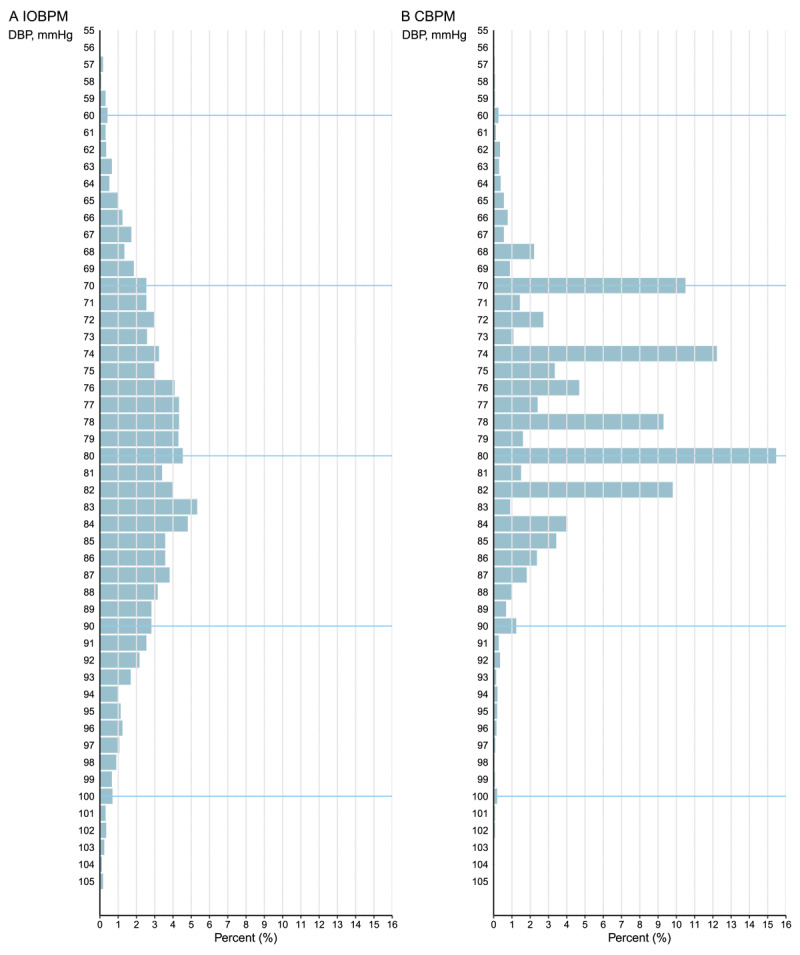
The comparison of diastolic blood pressure measurements. Abbreviations: IOBPM, intelligent office blood pressure measurement; CBPM, conventional blood pressure management; DBP, diastolic blood pressure.

Compared with the IOBPM model, the CBPM model preferred specific BP values. The most frequently recorded SBP values in the CBPM model were 130 mmHg (12.7%), 132 mmHg (11.9%), 126 mmHg (9.6%), and 120 mmHg (7.6%). The most frequently recorded DBP were 80 mmHg (15.5%), 74 mmHg (12.2%), 70 mmHg (10.5%), and 82 mmHg (9.8%). In contrast, BP readings in the IOBPM model showed a nearly normal distribution (Figures 3 and 4).

### Blood pressure control

The overall BP control was significantly higher in the CBPM model than in the IOBPM model (90.2% vs 42.0%, P < 0.001). In the IOBPM model, the BP control for males was higher than that for females (44.3% vs 40.1%, *P* < 0.001), and there was a trend of decreasing BP control with increasing age [age groups: 35–59 (50.3%), 60–79 (43.1%), 70–79 (39.5%), and 80–89 (38.3%); Z = 3.55, *P* for trend < 0.001]. Within the CBPM model, the BP control was higher in females than males (91.7% vs 88.7%, *P* = 0.02). The trend observed in the CBPM model was opposite to that in the IOBPM model, with a higher BP control associated with increasing age [age groups: 35–59 (88.9%), 60–79 (89.6%), 70–79 (91.3%), and 80–89 (90.3%); Z = –1.82, *P* for trend = 0.03] (Table 2).

**Table 2:** Blood pressure control of hypertensive patients.


VARIABLES	IOBPM		CBPM	
	
	n	%	n	%

Overall	2,909	42.0	5,744	90.2

Sex				

Male	1,324	44.3	2,604	88.5

Female	1,585	40.1	3,140	91.7

Age group (years)				

35–59	324	50.3	675	88.9

60–69	1,141	43.1	2,233	89.6

70–79	1,196	39.5	2,321	91.3

80–89	248	38.3	515	90.3


Abbreviations: IOBPM, intelligent office blood pressure measurement; CBPM, conventional blood pressure management; SBP, systolic blood pressure; DBP, diastolic blood pressure.

## Discussion

The data quality of overall BP measurements improved with the IOBPM model compared with the CBPM model. The IOBPM model showed higher BP levels, did not show preferences for end-digits for both SBP and DBP values, showed a nearly normal distribution of BP values without any preference for specific SBP and DBP values, and realistic BP control status.

Previous studies have demonstrated that BP levels increased and BP control decreased after implementing automated BP measurements (11,12). Our study confirms these findings, showing an increase in mean SBP value of 12.3 mmHg with the use of the IOBPM model compared to the CBPM model and a significant decrease in the hypertension control from 90.2% in the CBPM model to 42.0% in the IOBPM model. However, contrasting results have also been reported. The BP measurements achieved in a systolic blood pressure intervention trial (SPRINT), measured with unattended automated office blood pressure (UAOBP) devices, were, on average, lower than casual office measurements (13). A quality improvement project (14) compared office BP measured using the routine clinic visit protocol versus a standardized SPRINT-like protocol. The results showed that the mean blood pressure was 10.3/6.3 mmHg lower using the SPRINT-like UAOBP. This could be explained by several factors affecting the CBPM model, such as recall/reporting bias or a trend to report lower BP values by patients to their PCPs during the follow-up. This situation was also found in another study about self-reported BP readings by patients (15). The investigation in the Minhang district of Shanghai also found that approximately 73% of patients with hypertension were likely to share their HBPM readings with their PCPs, but most reported their results by recall instead of by notebook or machinery memory (16).

End-digit preference is a recognized indicator of erroneous BP recording associated with biased observations or fewer patients with elevated BP (11). Several studies found a strong preference for ‘zero’ as an end-digit in BP readings and showed that zero preference decreased using automatic BP devices (11,17,18,19). A retrospective observational study reviewed the trends in end-digit preference for BP in primary care facilities in Canada and the United Kingdom (UK) from 2006 to 2015 and found that the frequency of last digit zero for both SBP and DBP decreased over time by 11.2% in Canada and by 6.9% in the UK (20). Similarly, another study in Shanghai’s Minhang district (21) analysed the changes in end-digit preference of BP readings in primary clinics from 2007 to 2011 and found that end-digit preferences decreased from 62.1% to 47.6%. This trend was also seen in our study, and although our most common end-digit preference value was also ‘zero,’ our frequency was 20% lower than the results for zero preference within the Minhang district (21). Office BP measurements using automatic devices, either unattended or partly attended, can provide multiple BP readings and avoid the white-coat effect without observer error. Although the zero preference in the CBPM model was not as strong as in previous studies (17,18,20), there was still a significant end-digit preference compared with the BP readings in the IOBPM model. Our study indicated that the end-digit preference problem in BP measurements has improved in the last decade in Shanghai. This is attributable to recent BP device changes and quality improvements in primary hypertension management.

Similarly, certain preferred BP values were found in previous studies (11,17,18,19,20). We found the preferred BP values in the CBPM model were all below the threshold (140/90 mmHg). This could have been that the PCPs were attempting to meet the hypertension control targets in Shanghai. A study conducted in the United Kingdom found evidence of increased preference for the values just below pay-for-performance targets (22). In contrast, the normal distribution of BP readings in the IOBPM model was similar to the results in another study (11).

The proportion of BP control in the IOBPM model was higher than the control rate of hypertension in a large-scale national investigation during 2012–2015 (42.0% vs 37.5%) (3), but was similar to the control rate in urban areas of China (42.4%), and in other countries including USA, Costa Rica, Germany, and Portugal (23). A recent study published by our team compared the agreement among IOBP, awake ambulatory BP (ABP), and conventional auscultatory OBP at different BP levels. The results showed that BP measured using the IOBPM model was consistent with awake ABP and conventional OBP and can be a good choice in the Chinese community (24). This indicates that the control rate in the IOBPM model may better reflect the current hypertension control among patients being treated and managed in CHCs of Shanghai.

Applying the new IOBPM model at pilot CHCs could improve the BP data quality in the routine follow-up and management of hypertensive patients and prevent clinical therapeutical inertia and higher cardiovascular event risk. Multiple office BP measurements have been recommended by the guidelines, but the standard operating procedures for office BP measurements vary greatly (6). The UAOBP model (25) was suggested based on several advantages, such as less digit preference, consistent readings between visits, the reduction of the white coat effect, and reduced workload for HCPs (26,27). However, the disadvantage of requiring more resources (separate room for measurement and more time for the procedure), is that attended office BP measurement can be applied more readily in general practice and hospital clinic environments (28). In the IOBPM model, one or two HCPs were present to assist the primarily elderly patients during the BP measurement. We followed the Chinese hypertension guidelines (8) and used the average of two or three measurements, instead of the average of the last two of three readings recommended by the recently published European hypertension guidelines (29). In addition, the IOBPM was also based on the findings from the cohort study (6) in Shanghai’s Minhang district that identified calculating BP as the average of two or three consecutive BP measurements was more accurate in predicting all-cause and cardiovascular mortality than the average of the last two or the mean of the three readings. Using a standardized recording and transmission system allows physicians to assess all available BP values and automatically calculate the average BP, which could help to determine whether antihypertensive medications should be modified.

### Strengths and Limitations

The main strength of the current study is the piloting of the IOBPM model in clinical practice. We believe that introducing this model will improve the accuracy of current BP measurements, even if taking the IOBPM model to scale-up in clinical settings may take time as HCPs and patients adapt to the new practice. The major limitation was that we couldn’t differentiate between the office and home BP measurements in the CBPM model, as we did not have information about the BP devices or BP measurement methods, i.e., manual or automatic devices, or whether the measurements were taken in the office or self-reported as HBPM. As the IOBPM model was not stratified by age groups, there was a selection bias with people in the 35–59 age group being underrepresented, and women with higher-education levels being overrepresented. For the CBPM model group, there was also a selection bias because only patients who had BP records in the NCDs information system within the most recent three months were selected, whereas those who missed their follow-up visit were excluded. We also did not have the hypertension duration of patients in the CBPM model for the comparison. Finally, although our population is representative of patients with hypertension under the management in CHCs in Shanghai, the results cannot be extrapolated to other Chinese cities.

## Conclusions

The IOBPM model appears to overcome the deficiencies observed with the CBPM model, leading to more accurate and reliable BP measurements. We believe the IOBPM model should be adopted as the standard for community-based hypertension management and extended to all the CHCs in Shanghai and other cities in China. Exploring the difference in the control rate and cardiovascular event incidence between the two models should be the topic of future research.

## Data Accessibility Statement

The data underlying this article will be shared on reasonable request to the corresponding author.
